# Hurricanes and Health: A Scoping Review of Recent Developments in Physical Injuries, Mental Health, and Emergency Interventions

**DOI:** 10.7759/cureus.63973

**Published:** 2024-07-06

**Authors:** Micah Ngatuvai, Tanner C Blaylock, Rachel Ngatuvai, Landon Saipale, Gary Schwartz

**Affiliations:** 1 College of Allopathic Medicine, Nova Southeastern University Dr. Kiran C. Patel College of Allopathic Medicine, Fort Lauderdale, USA; 2 Orthopedics, William Beaumont Army Medical Center, El Paso, USA; 3 Orthopedics, Texas Tech University Health Sciences Center El Paso, El Paso, USA; 4 Trauma Intensive Care Unit, Memorial Healthcare System, Fort Lauderdale, USA; 5 College of Medicine, Central Michigan University, Saginaw, USA; 6 Orthopaedic Surgery, Nova Southeastern University Dr. Kiran C. Patel College of Allopathic Medicine, Fort Lauderdale, USA

**Keywords:** emergency medical services, social determinants of health, disaster management, covid-19 interventions, mental health outcomes, physical injuries, public health impacts, natural disasters, hurricane

## Abstract

Hurricanes, as one of the most devastating natural disasters, significantly impact the public's health, causing both physical injuries and long-lasting mental health issues. Although substantial research has focused on hurricane-related injuries, this study aims to synthesize findings from recent literature, specifically evaluating the 10 most recent hurricanes, to identify research gaps and inform future studies.

This scoping review, conducted in accordance with PRISMA-Scr guidelines, assessed studies from PubMed, CINAHL, Cochrane databases, and Medline as of February 2024. Eligibility criteria focused on studies examining physical and mental health impacts, COVID-19 effects, and emergency medical services (EMS) interventions related to Hurricanes Ian, Nicholas, Ida, Zeta, Delta, Sally, Laura, Isaias, Hanna, and Dorian.

Twenty articles met the inclusion criteria. The studies were categorized into four themes: physical injuries and fatalities, mental health impacts, hurricane-COVID-19 interplay, and EMS interventions. Findings revealed varied mechanisms of injuries and deaths, significant mental health challenges, compounded crises due to COVID-19, and diverse EMS strategies, including AI utilization and strategic planning for medical care delivery.

Addressing the social determinants of health and evaluating hurricane readiness initiatives were two gaps in the literature identified. Future research should focus on the mental health impacts and concurrent crisis challenges to develop comprehensive disaster management practices that enhance community resilience against future hurricanes and public health crises.

## Introduction and background

Hurricanes are among the most devastating natural disasters, which have caused approximately 6,890 deaths since 1980, with an average cost of $22.8 billion per event [1]. Every year, these powerful storms cause massive destruction and disruption of daily life. These storms not only cause extensive physical destruction but also profoundly impact public health. The effects of these storms include a wide-ranging spectrum of injuries, from minor lacerations to life-threatening conditions, leading to long-lasting mental health issues in affected communities [2].

Research on hurricane-related injuries has been an area of active investigation, yielding insights into various aspects of these injuries [2-4]. Studies have delved into specific hurricanes and explored the mechanisms of injury, including those related to shutters and trees [2-4]. Other studies have explored the psychological impacts of hurricanes, including post-traumatic stress disorder (PTSD) and other mental health issues following a hurricane [5]. Given the ongoing research into hurricane-related injuries and their psychological impacts, there is a critical need to synthesize this body of work to identify gaps and guide future investigations more effectively.

This scoping review targets this need by evaluating the literature concerning the 10 most recent hurricanes identified by the National Oceanic and Atmospheric Administration (NOAA) with a focus on physical injuries, mental health outcomes, and the efficacy of intervention strategies [6]. By incorporating recent studies, this review aims to present an updated overview reflecting current trends, challenges, and advancements in managing the health impacts of hurricanes. Our objective is not only to summarize existing knowledge but also to spotlight areas lacking research, thereby providing a structured path for future studies.

## Review

Methods

This scoping review was conducted following the Preferred Reporting Items for Systematic Reviews and Meta-Analyses Extension for Scoping Review (PRISMA-Scr) guidelines (Appendix 1), incorporating additional best practices from Peters et al. when necessary [7,8]. Prior to initiation, this study was submitted to the Institutional Review Board (IRB) and deemed exempt. This study was also pre-registered with the Open Science Framework (OSF) in order to reduce bias [9]. A comprehensive search was conducted on February 15th across PubMed, CINAHL, Cochrane databases, and Medline, targeting studies on physical or mental health injuries resulting from hurricanes or interventions designed to address their impacts.

Eligibility Criteria

We predefined eligibility criteria, detailed on OSF, focusing on studies related to the 10 most recent hurricanes as reported by NOAA and made available on their website as of February 2024. Specifically, these included Hurricanes Ian, Nicholas, Ida, Zeta, Delta, Sally, Laura, Isaias, Hanna, and Dorian. These hurricanes occurred from August 2019 to September 2022. Included studies were those examining injury types and mechanisms, mortality, mental health impacts, the effects of COVID-19, or the use of emergency medical services (EMS) post-hurricane. Additional considerations included health interventions aimed at affected populations. Articles were excluded if they were non-English, were not primarily focused on the aforementioned hurricanes, or did not contain sufficient information for analysis. Book reviews, correspondence, erratum, and conference posters or abstracts were also excluded.

Search Strategy

An electronic search of PubMed, CINAHL, Cochrane databases, and Medline was conducted to identify studies related to hurricane injuries, using the Boolean phrase: "((((((((((Hurricane Ian) OR (Hurricane Nicholas)) OR (Hurricane Ida)) OR (Hurricane Zeta)) OR (Hurricane Delta)) OR (Hurricane Sally)) OR (Hurricane Laura)) OR (Hurricane Isaias)) OR (Hurricane Hanna)) OR (Hurricane Dorian))” and was conducted for all mentioned databases. Additionally, a secondary search of all references to the included studies was assessed for eligibility.

Identification of Studies and Data extraction

**Figure 1:**
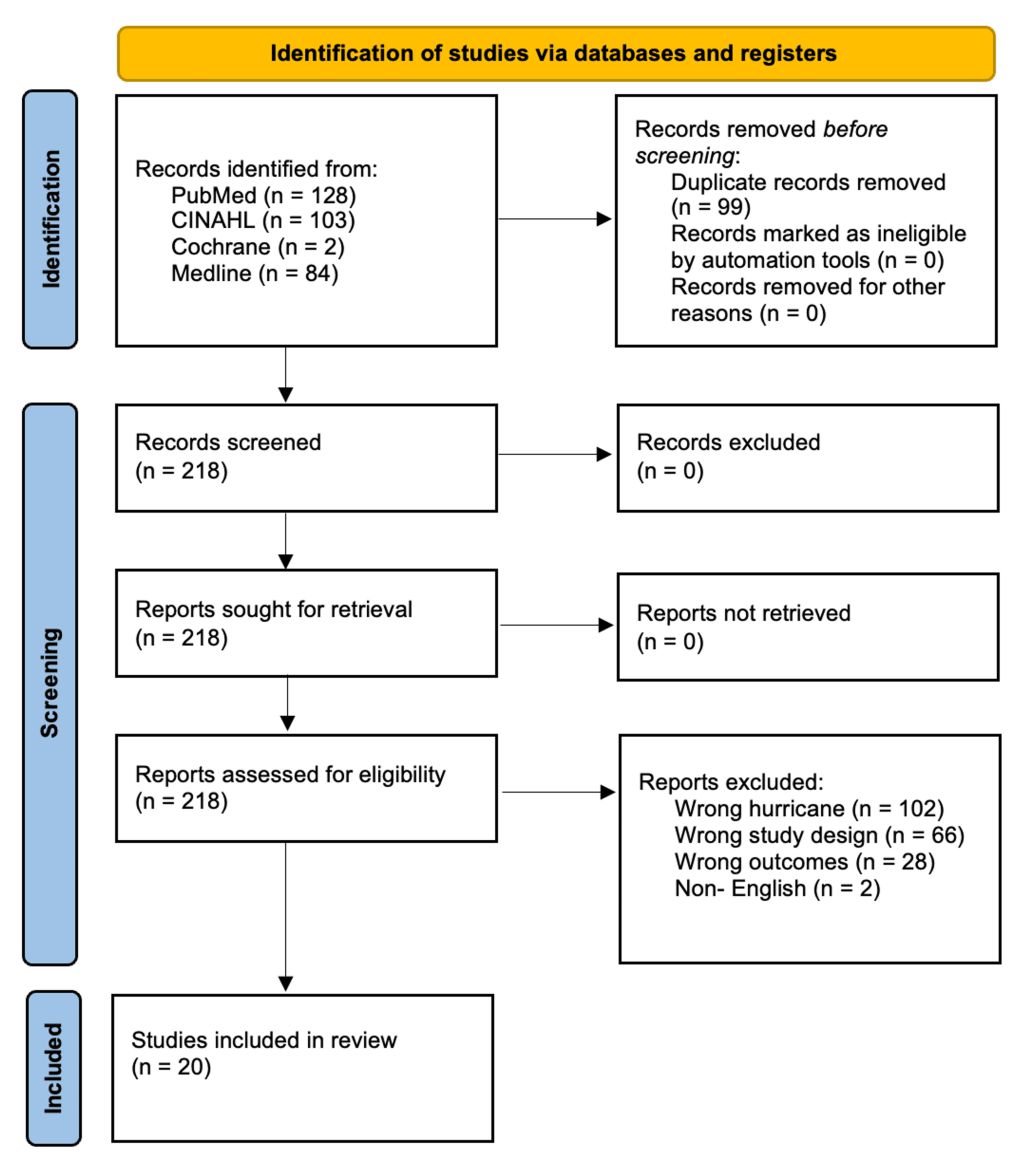
This PRISMA flow diagram illustrates the selection process for the studies included.

The Boolean and keyword searches resulted in 317 articles, removing 99 duplicates. We screened the remaining articles for relevance based on our criteria, of which 20 were found to be eligible (Figure 1). Two reviewers independently conducted the selection process, with discrepancies resolved through consultation of a third reviewer and with discussion of the predefined eligibility criteria.

Results

Our review found 20 eligible studies that were categorized into distinct themes: physical injuries and fatalities, mental health impacts, the interplay between hurricanes and COVID-19, and the efficacy of EMS and interventions (Table 1) [10-29].

**Table 1:** A description of each study which includes: Author, hurricane, location, study type, and main study findings. EMT: Emergency medical technician, NDMS: National disaster medical system, AI: Artificial intelligence, VA: Veteran affairs

Author (year)	Hurricane	Location	Study Type	Findings
Dunne-Sosa et al. (2019) [14]	Dorian	Bahamas	Field Report	Healthcare infrastructure needs to be improved to treat conditions such as mental health and withstand emergency situations.
Randolph et al. (2019) [26]	Dorian	United States	Field Report	Heart to Heart International showed logistical success when operating in austere environments.
Bouland et al. (2019) [25]	Dorian	United States	Field Report	Faster access of mobile EMT groups to communities’ post hurricane improves impact of health services provided.
Louissaint (2019) [27]	Dorian	United States	Field Report	Hurricanes lead to significant destruction of public health and healthcare infrastructure but lead to opportunities to build back better.
Garrett (2020) [22]	Laura	United States	Field Report	The NDMS implemented adaptations to their typical medical disaster response protocol to mitigate risks of the on-going COVID-19 pandemic.
Shultz et al. (2020) [12]	Dorian	Bahamas	Review	Mental health needs to be continually focused with resources provided in times of natural disaster.
Hanchey et al. (2021) [11]	Ida	United States	Field Report	Cumulative death counts and causes across nine states.
Chang et al. (2021) [29]	Ida	United States	Viewpoint	Teamwork and collective resilience is necessary in the face of disaster.
Tracey et al. (2022) [16]	Laura	United States	Observational Study	Hurricane Laura increased Covid-19 transmission, especially in low-income areas.
Page-Tan et al. (2022) [20]	Zeta	United States	Cross Sectional	Hotel accommodations among other measures may help with maintaining COVID-19 protocols.
Bathina et al. (2022) [13]	Multiple	United States	Longitudinal Study	Monitoring social media after hurricanes can give insight into community improvement.
Shultz et al. (2022) [18]	Ida	United States	Viewpoint	COVID-19 adds to the complexities of hurricane management.
Fenton et al. (2022) [28]	Ida	United States	Field Report	Hurricane Ida emphasized the need for strategic planning improvement for an organized university program.
Wertis et al. (2023) [15]	Ida	United States	Longitudinal	More mental health support is needed for those impacted by hurricanes.
Cao (2023) [23]	Ian	Australia	Viewpoint	The use of AI for smart disaster resilience improves the typical organized responses to natural disasters.
Greer et al. (2023) [17]	Laura	United States	Observational	COVID-19 had indirect but not direct effects on a person's evacuation decision making for hurricane Laura.
Wu et al. (2023) [19]	Laura	United States	Observational	Risk perceptions of hurricane impacts, and COVID-19 significantly impact hurricane evacuation decisions.
Girard et al. (2023) [21]	Laura	United States	Cross Sectional	Hurricane Laura significantly impacted individuals’ ability to adhere to COVID-19 precautions.
Bushong et al. (2023) [10]	Ian	United States	Review	The Older white population was most affected and indirect deaths outnumbered direct death causes.
Haverhals et al. (2024) [24]	Ian	United States	Observational Study	VA staff preparedness pre, during, and post-hurricane contributed to positive mental health outcomes in elderly.

Physical Injuries and Deaths

Two studies were included assessing the impact of hurricanes on physical injuries and fatalities. The first study delineated three categories of storm-related fatalities post-Hurricane Ian: direct, indirect, and unrelated to the storm. Direct fatalities were primarily due to drowning in storm surges, while indirect deaths involved older adults lacking critical life-support resources like dialysis and oxygen due to power outages and infrastructure damage [10]. The second study focused on Hurricane Ida, reported 91 deaths across nine states, with drowning being the most common cause, particularly in the Northeast [11]. It emphasized the varied circumstances leading to fatalities, including vehicular incidents and generator or power outage-related issues.

Mental Health

Four studies focusing on mental health outcomes of hurricane-affected populations were found. The first study focused on Hurricane Dorian, illustrating the critical need for continuity of care and access to medications for individuals with chronic conditions, affecting mental health indirectly through the exacerbation of pre-existing conditions [12]. The second study analyzed social media to quantify emotional responses to hurricanes, revealing significant shifts in societal sentiment and the importance of digital platforms in understanding real-time mental health needs [13]. A third study on Hurricane Dorian’s mental health consequences emphasized the acute stress responses among survivors, showcasing the immediate and lingering psychological effects [14]. Lastly, an analysis of Hurricane Ida’s impact demonstrated a surge in mental health crisis communications, underscoring the increased demand for mental health services post-disaster [15].

Hurricane Response Amidst COVID-19

A total of six studies were found that navigate the intricate dynamics between hurricane response and COVID-19 management, offering diverse perspectives on this dual crisis. The first study evaluated an evacuation's impact on COVID-19 spread and suggested mitigating shelter strategies [16]. The second research study investigated households' evacuation decisions during Hurricane Laura as impacted by COVID-19 [17]. Another study focused on Hurricane Ida, emphasizing the compounded health system challenges [18]. One analysis discussed the struggle to maintain social distancing during evacuations [19]. Another highlighted the disregard for COVID-19 precautions in hurricane shelters [20]. The final study emphasized the importance of hand hygiene practices in mitigating virus spread during such crises, advocating for public health adaptability [21]. Together, these studies illuminate the multifaceted challenges and necessary strategic responses at the intersection of pandemic and natural disaster management.

EMS Services and Hurricane Interventions

Eight articles explored Emergency Medical Services (EMS) and interventions during hurricane disasters. One study outlined the utilization of the Federal Emergency Management Agency (FEMA), which organized several disaster medical assistance teams (DMATs) during Hurricane Laura and detailed the use of patient triaging and diagramming logistics to reduce COVID-19 risks [22]. Another highlighted the crucial role of AI in boosting disaster resilience through data-driven crisis management [23]. A separate investigation focused on the experiences of veterans and caregivers during Hurricane Ian, stressing the importance of preparedness [24]. Others focused on the impact of Hurricane Dorian on Eastern North Carolina and the Bahamas, underscoring the need for community rebuilding [25-27]. A publication about a university's emergency medicine response to Hurricane Ida demonstrated the necessity of strategic planning, echoed by a medical department's efforts in similar conditions [28,29]. Of the studies, post-Hurricane Dorian, one in particular regarding an international organization's logistical success in the Bahamas exemplified effective medical care delivery [26]. Finally, a review of a rapid response team's deployment to the Bahamas after Hurricane Dorian showcased the significance of readiness in disaster response, summarizing a broad analysis of disaster management strategies [25].

Discussion

To the best of our knowledge, this scoping review is the first to evaluate the effects of hurricanes on physical injuries, mental health, and the role of Emergency Medical Services (EMS) and related interventions. It compiles and analyzes the spectrum of literature on the health repercussions of recent hurricanes, revealing important trends of research investigating physical injuries and fatalities, mental health challenges, hurricane management in the wake of COVID-19, and the adaptive strategies of EMS. This analysis not only reaffirms the acute and long-term public health challenges posed by hurricanes but also brings to light the evolving complexities of disaster management in the face of concurrent crises such as the COVID-19 pandemic.

Hurricane impacts on physical injury types and mechanisms have been extensively studied and continue to be an area of focus [2-4]. However, our review illuminates a noticeable shift towards more detailed investigations into mental health outcomes, signifying an increased recognition of psychological resilience and vulnerability in disaster-stricken populations. This trend reflects a broader understanding of disaster impacts, extending beyond immediate physical injuries to include long-term psychological well-being.

Notably, our findings regarding the intersection of hurricanes and the COVID-19 pandemic reveal a novel and complex challenge in public health management. The dual crisis exacerbates the vulnerability of affected communities, highlighting significant gaps in current emergency preparedness and response frameworks. The studies reviewed suggest the need for innovative approaches to disaster management that are adaptable to the multifaceted nature of concurrent crises [17,19]. These findings advocate for integrated disaster response strategies that encompass not only immediate medical and logistical support but also comprehensive public health planning that anticipates and mitigates the compounded effects of overlapping disasters, such as a worldwide pandemic and regional natural disasters.

The discussion on EMS services and hurricane interventions reflects a diverse array of tactics employed in disaster response, underscoring the critical role of adaptability and preparedness. From the utilization of AI in crisis management to the strategic planning required for medical care delivery in disaster contexts, these insights underscore the necessity for a multifaceted approach to disaster management [22,23]. Moreover, the exploration of these strategies through the lens of recent hurricanes offers valuable lessons for enhancing resilience and response capabilities in future events.

This review calls for an intensified focus on mental health research in the aftermath of hurricanes, urging future studies to delve deeper into the mechanisms of psychological impact and resilience. Additionally, the intersection of hurricanes and COVID-19 prompts a reevaluation of disaster preparedness and response strategies by local EMS, advocating for more agile, integrated approaches capable of addressing the multifaceted challenges of concurrent crises. Furthermore, our study found a significant gap concerning the outcomes related to social determinants of health, such as socioeconomic status, which could provide critical insights into the disparities in impact and recovery. Equally, there is an apparent absence of studies investigating the efficacy of specific preparation measures for hurricanes, underscoring the need for dedicated research to enhance pre-disaster readiness and mitigate adverse outcomes.

Our review is not without limitations. The exclusion of non-English language studies and the focus on only the most recent hurricanes narrowed the scope of our findings to include only named hurricanes with North American landfall from August 2019 to September 2022 while excluding impacts of named tropical storms during the same time period. This limitation may affect the generalizability of our findings, potentially introducing a geographical and temporal bias that could overlook significant data from older or non-English sources. Furthermore, the inclusion of direct reports from the field, which were not subjected to the rigorous peer review process typical of academic research, might affect the overall strength of evidence and robustness of the conclusions drawn.

Despite these limitations, the deliberate focus on recent hurricanes ensures that our findings are highly relevant to the current state of disaster response and preparedness. By highlighting these specific constraints, we aim to underscore areas for methodological enhancement and provide a framework for future research studies aimed at the improvement of public health and emergency management.

## Conclusions

This scoping review categorized the included articles into four main categories: physical injuries, mental health, COVID-19 challenges, and EMS interventions. These categories illustrate the complex layers of impact that hurricanes exert on health and emergency systems, particularly under the compounded pressures of the concurrent COVID-19 pandemic. The review underscores an urgent need for a paradigm shift in disaster management strategies, highlighting that traditional methods may fall short in addressing the simultaneous impacts of overlapping crises.

Two critical gaps identified in the literature were the impact of social determinants on health outcomes during hurricanes and the effectiveness of hurricane readiness initiatives, including meteorological warnings and emergency preparedness programs for both volunteers and professionals. Addressing these gaps is vital for enhancing disaster resilience and ensuring effective response strategies. Additionally, continued research into mental health impacts and the challenges posed by pandemics like COVID-19 may be beneficial. These areas of research are crucial for developing holistic and adaptable disaster management practices that can safeguard communities against future hurricanes and concurrent public health crises.

## Appendices

**Table 2:** Appendix 1: PRISMA guideline checklist JBI: Joanna Briggs Institute; PRISMA-ScR: Preferred Reporting Items for Systematic Reviews and Meta-Analyses Extension for Scoping Reviews. * Where sources of evidence (see second footnote) are compiled from, such as bibliographic databases, social media platforms, and Web sites. † A more inclusive/heterogeneous term used to account for the different types of evidence or data sources (e.g., quantitative and/or qualitative research, expert opinion, and policy documents) that may be eligible in a scoping review as opposed to only studies. This is not to be confused with information sources (see first footnote). ‡ The frameworks by Arksey and O’Malley (6) and Levac and colleagues (7) and the JBI guidance (4, 5) refer to the process of data extraction in a scoping review as data charting. § The process of systematically examining research evidence to assess its validity, results, and relevance before using it to inform a decision. This term is used for items 12 and 19 instead of "risk of bias" (which is more applicable to systematic reviews of interventions) to include and acknowledge the various sources of evidence that may be used in a scoping review (e.g., quantitative and/or qualitative research, expert opinion, and policy document).

SECTION	ITEM	PRISMA-ScR CHECKLIST ITEM	REPORTED ON PAGE #
TITLE			
Title	1	Identify the report as a scoping review.	Pg 1
ABSTRACT			
Structured summary	2	Provide a structured summary that includes (as applicable): background, objectives, eligibility criteria, sources of evidence, charting methods, results, and conclusions that relate to the review questions and objectives.	Pg 2
INTRODUCTION			
Rationale	3	Describe the rationale for the review in the context of what is already known. Explain why the review questions/objectives lend themselves to a scoping review approach.	Pg 2-3
Objectives	4	Provide an explicit statement of the questions and objectives being addressed with reference to their key elements (e.g., population or participants, concepts, and context) or other relevant key elements used to conceptualize the review questions and/or objectives.	Pg 2-3
METHODS			
Protocol and registration	5	Indicate whether a review protocol exists; state if and where it can be accessed (e.g., a Web address); and if available, provide registration information, including the registration number.	Pg 4-5
Eligibility criteria	6	Specify characteristics of the sources of evidence used as eligibility criteria (e.g., years considered, language, and publication status), and provide a rationale.	Pg 4-5
Information sources*	7	Describe all information sources in the search (e.g., databases with dates of coverage and contact with authors to identify additional sources), as well as the date the most recent search was executed.	Pg 4-5
Search	8	Present the full electronic search strategy for at least 1 database, including any limits used, such that it could be repeated.	Pg 4-5
Selection of sources of evidence†	9	State the process for selecting sources of evidence (i.e., screening and eligibility) included in the scoping review.	Pg 4-5
Data charting process‡	10	Describe the methods of charting data from the included sources of evidence (e.g., calibrated forms or forms that have been tested by the team before their use, and whether data charting was done independently or in duplicate) and any processes for obtaining and confirming data from investigators.	Pg 4-5
Data items	11	List and define all variables for which data were sought and any assumptions and simplifications made.	Pg 4-5
Critical appraisal of individual sources of evidence§	12	If done, provide a rationale for conducting a critical appraisal of included sources of evidence; describe the methods used and how this information was used in any data synthesis (if appropriate).	NA
Synthesis of results	13	Describe the methods of handling and summarizing the data that were charted.	Pg 4-5
RESULTS			
Selection of sources of evidence	14	Give numbers of sources of evidence screened, assessed for eligibility, and included in the review, with reasons for exclusions at each stage, ideally using a flow diagram.	Pg 5-8
Characteristics of sources of evidence	15	For each source of evidence, present characteristics for which data were charted and provide the citations.	Pg 5-8
Critical appraisal within sources of evidence	16	If done, present data on critical appraisal of included sources of evidence (see item 12).	NA
Results of individual sources of evidence	17	For each included source of evidence, present the relevant data that were charted that relate to the review questions and objectives.	Table 1
Synthesis of results	18	Summarize and/or present the charting results as they relate to the review questions and objectives.	Pg 5-8, Table 1
DISCUSSION			
Summary of evidence	19	Summarize the main results (including an overview of concepts, themes, and types of evidence available), link to the review questions and objectives, and consider the relevance to key groups.	Pg 8 to 10
Limitations	20	Discuss the limitations of the scoping review process.	Pg 8 to10
Conclusions	21	Provide a general interpretation of the results with respect to the review questions and objectives, as well as potential implications and/or next steps.	Pg 10
FUNDING			
Funding	22	Describe sources of funding for the included sources of evidence, as well as sources of funding for the scoping review. Describe the role of the funders of the scoping review.	Pg 10
